# The introduction of balint groups for core medical trainees – a pilot

**DOI:** 10.1192/bjo.2021.358

**Published:** 2021-06-18

**Authors:** Itunuayo Ayeni, Anne Patterson

**Affiliations:** Central and North West London NHS Foundation Trust

## Abstract

**Aims:**

To introduce and assess the impact of balint groups on core medical trainee (CMT) doctors working within an acute medical trust.

**Background:**

A high rate (80%) of dissatisfaction and burnout has been reported amongst trainee doctors. This has had a significant impact on recruitment with a large proportion of foundation doctors delaying their application into core specialist training. Of those already in training, up to 50% have reported taking time, out citing burnout as a cause. Balint groups are a form of reflective practice groups looking at the doctor-patient interaction. For core psychiatric trainees these groups are a mandatory part of their training.

**Method:**

We piloted a total of three balint groups over a period of three months amongst CMT doctors based at an acute medical trust in London. A specialty registrar (ST6) in psychiatry facilitated the balint groups. Balint facilitators received supervision from a consultant psychiatrist in psychotherapy. CMT doctors were given questionnaires at the beginning of session one and emerging themes later explored. The questionnaires used were taken from the ‘Bristol Trainee-led Balint Group Scheme’.

**Result:**

The pre-questionnaires showed that all CMT doctors surveyed believed psychological factors play an important role on patient presentation and recovery. 14/19 (74%) agreed or strongly agreed that a doctor's reaction to a patient directly influenced care. All doctors agreed or strongly agreed that it was important to reflect on a patient's emotional experience, as it was crucial to their development as a doctor.

CMT doctors found balint groups useful as it provided them a space, which was not routinely offered to discuss challenging cases. Themes that emerged included a lack of support and difficulties maintaining boundaries when treating complex patients. Litigation was a recurring theme with many trainees reporting anxieties and a lack of support. Trainees reported guilt and worries that they were not doing enough for their patients. These themes appeared to have a direct impact on training experience and burnout.

**Conclusion:**

With increasing burnout and dissatisfaction amongst junior doctors, balint groups provide a unique approach to supporting junior doctors within medical specialties. The current pilot has demonstrated that CMT doctors can make use of balint groups in an effective way. We recommend that balint groups should become an integral part of specialist medical training. Psychiatrists can play a central role in supporting the health and well being of medical trainees through balint group facilitation.

